# Beyond Blood Sugar: Investigating the Cardiovascular Effects of Antidiabetic Drugs

**DOI:** 10.7759/cureus.46373

**Published:** 2023-10-02

**Authors:** Binish A Ahmad, Isha M Sanghani, Rithika Sayabugari, Hannah Biju, Akshaya Siddegowda, Minnu Ittiachen Kinattingal, Sai Goutham Reddy Yartha, Prajyoth M Gaonkar, Syed Shireen Andrabi, Yogeshkumar K Vaghamashi, Arunika Korwar

**Affiliations:** 1 Department of Internal Medicine, King Edward Medical University, Lahore, PAK; 2 Department of Internal Medicine, Punyashlok Ahilyadevi Holkar Government Medical College, Baramati, IND; 3 Department of General Medicine, Gandhi Medical College, Hyderabad, IND; 4 Department of Internal Medicine, Kristu Jayanti College, Bengaluru, IND; 5 Department of Internal Medicine, JSS Medical College, Mysuru, IND; 6 Department of Internal Medicine, New Hope Clinical Research, Charlotte, USA; 7 Department of Internal Medicine, Karuna Medical College, Palakkad, IND; 8 Department of Internal Medicine, Sri Venkateswara Medical College, Tirupati, IND; 9 Department of Internal Medicine, School of Medicne, Tehran University of Medical Sciences, Tehran, IRN; 10 Department of Internal Medicine, Bicol Christian College of Medicine, Legazpi, PHL; 11 Department of Internal Medicine, KJ Somaiya Medical College, Mumbai, IND

**Keywords:** glp-1 agonist, sglt-2 inhibitor, tirzepatide, insulin, anti-diabetic drugs

## Abstract

Cardiovascular disease is a major comorbidity associated with diabetes mellitus. Various antidiabetic drugs are currently used to treat type 2 diabetes mellitus and have varying effects on the cardiovascular system. Some drugs, such as glucagon-like peptide 1 (GLP-1) agonists and sodium-glucose cotransporter 2 (SGLT-2) inhibitors, are cardioprotective, whereas others, such as insulin, have deleterious effects on the cardiovascular system. This narrative review assessed the impact of antidiabetic drugs on cardiovascular health in the management of diabetes mellitus. It critically examines various classes of these medications, including conventional options such as metformin and newer agents such as incretin-based therapies and SGLT-2.

## Introduction and background

Diabetes mellitus, commonly referred to as DM, is a metabolic condition characterized by elevated levels of blood glucose. Insulin helps stabilize blood glucose levels by promoting cellular uptake of glucose and inhibiting hepatic gluconeogenesis [1]. DM is caused either by insufficient production or a lack of response to the hormone insulin [1]. The International Diabetes Federation reports that approximately 415 million individuals globally are believed to have diabetes, and it is estimated that type 2 diabetes mellitus (T2DM) accounts for approximately 91% of these cases [2]. The prevalence of T2DM has consistently increased [2]. Diabetic complications account for 7-10% of all fatalities worldwide [3]. Individuals diagnosed with DM have a heightened susceptibility to the development of various complications, including macrovascular (cardiovascular disease, peripheral vascular disease, cerebrovascular accident) and microvascular (retinopathy, nephropathy) complications [4]. In addition to vascular diseases, DM also poses a risk for non-vascular conditions, such as cancer and premature death [4]. Cardiovascular complications are the main factors that contribute to the morbidity and mortality associated with diabetes. Over 75% of people with diabetes over the age of 40 years die from cardiovascular disease [5]. T2DM poses a considerable risk for the development of coronary artery disease (CAD) and is frequently considered to be at par with established CAD risks [5]. Around 50% of individuals diagnosed with T2DM go on to develop heart failure [6].

Regulation of blood glucose levels involved in the medical management of T2DM treats short-term metabolic effects and prevents long-term vascular complications [7]. Metformin is considered the first-choice antidiabetic drug for glycemic control in patients with T2DM. This may improve the outcomes of cardiovascular diseases [8]. According to large randomized trials, significant reductions were observed in cardiovascular events for two sodium-glucose cotransporter 2 (SGLT2) inhibitors (empagliflozin and canagliflozin) and many glucagon-like peptide 1 (GLP-1) receptor agonists, such as semaglutide, dulaglutide, albiglutide, and liraglutide [6,9]. Despite substantial evidence from extensive cardiovascular outcome trials supporting the cardiovascular safety of dipeptidyl peptidase-4 (DPP-4) inhibitors, no benefit in preventing cardiovascular disease has been observed. In addition, saxagliptin, which belongs to the class of DPP-4 inhibitors, exhibited a higher incidence of hospitalization due to heart failure [3]. Evidence suggests that certain antidiabetic medications are associated with a higher risk of negative cardiovascular events. In a study conducted by Nissen et al, it was observed that treatment with rosiglitazone significantly elevated the risk of myocardial infarction and mortality from cardiovascular causes when compared to placebo or alternative forms of antidiabetic treatments [10].

The objective of this narrative review was to comprehensively explore and analyze the impact of various antidiabetic drugs on cardiovascular health, shedding light on their potential benefits and risks within the realm of diabetes management.

## Review

Biguanides 

Biguanides are used to manage diabetes, and they have demonstrated significant anti-cancer properties in both laboratory and clinical studies [11]. Among the biguanides, metformin has been widely prescribed for diabetes treatment since the 1950s. Buformin and phenformin, two drugs of the same class, were withdrawn in 1970 because they caused lactic acidosis [12]. Metformin is highly effective in treating diabetes due to its ability to lower blood sugar levels. It achieves this by reducing the production of glucose in the liver, facilitating glucose absorption in muscles, and promoting the breakdown of fatty acids in adipose tissue [13]. Metformin offers the advantage of promoting weight loss and poses no increased risk of hypoglycemia, thereby enhancing glycemic control [14]. 

Metformin has been shown to have multiple mechanisms of action. It can act through both AMP-activated protein kinase (AMPK)-dependent and AMPK-independent pathways. One mechanism involves inhibiting mitochondrial respiration, while another possibility is the inhibition of mitochondrial glycerophosphate dehydrogenase. Additionally, there seems to be a mechanism involving the lysosome [15]. Metformin has been shown to have favorable effects on blood pressure and plasma lipoprotein levels by reducing low-density lipoprotein cholesterol levels and potentially increasing high-density lipoprotein cholesterol [16].

Cardiovascular Effects

Metformin has been found to have positive effects on endothelial oxidative stress levels and inflammation caused by hyperglycemia, leading to a reduction in the occurrence of cardiovascular disease [17]. It achieves this by activating AMPK, which helps prevent alpha-dicarbonyl-mediated modifications of apolipoprotein residues. Consequently, metformin improves the function of high-density lipoproteins, reduces modifications in low-density lipoproteins, and enhances cholesterol transport. These improvements contribute to a decrease in cardiovascular risks [18]. A 20-year randomized multicenter trial called the United Kingdom Prospective Diabetes Study (UKPDS) demonstrated clear evidence that metformin therapy significantly lowers all-cause mortality and diabetes-related death over a ten-year period, particularly in overweight patients with T2DM [19]. A clinical study discovered that individuals with diabetes and confirmed atherothrombosis who received treatment with metformin experienced reduced rates of mortality compared to those who did not receive such treatment. According to observational data from the Registration, Evaluation, Authorisation and Restriction of Chemicals (REACH) Registry, using metformin as a secondary preventative measure was significantly linked to a 24.0% reduction in all-cause mortality after a 2-year follow-up [20]. According to a meta-analysis by Han et al., metformin demonstrated significant benefits in reducing cardiovascular mortality, all-cause mortality, and adverse cardiovascular events among individuals diagnosed with both coronary artery disease and T2DM [21]. However, the study did not observe any reduction in cardiovascular events for patients who did not have T2DM [21].

Adverse Effects

Metformin is known to cause significant gastrointestinal discomfort, including symptoms such as nausea, vomiting, and diarrhea. However, these adverse effects can be managed through careful adjustment of the dosage, effective communication with patients about potential side effects, and by utilizing extended-release versions of the medication. Metformin also causes Vitamin B12 deficiency, and lactic acidosis in patients with certain risk factors making them predisposed to lactic acidosis. It is worth noting that metformin boasts a strong safety profile and its affordability gives it an edge over other treatment options [19,22].

Sulfonylureas

Sulfonylureas are drugs with a phenyl-sulfonyl-urea structure responsible for its glucose-lowering effect [23,24]. There are two classes of sulfonylurea drugs, tolbutamide and chlorpropamide, which are first-generation drugs, and glyburide, glimepiride, and glipizide, which are second-generation sulfonylurea drugs [25]. Adenosine triphosphate (ATP)-sensitive potassium (K+) channels on pancreatic beta cells are bound to and inhibited by sulfonylureas, leading to depolarization and increased intracellular calcium, causing pancreatic beta cells to secrete more insulin [23,26]. Serum glucose levels can also be decreased by sulfonylureas by slowing down the metabolism of insulin in the liver, increasing the sensitivity of peripheral tissues to insulin, and limiting the liver's production of glucagon [27].

Cardiovascular Effects

It is widely recognized that individuals with T2DM who effectively control their blood sugar levels have a reduced likelihood of developing vascular damage. However, there is also evidence that suggests sulfonylurea drugs may be linked to an increased incidence of cardiovascular events due to the presence of SUR2A receptors on cardiac myocytes [28,29]. Numerous studies have indicated a potential link between the use of sulfonylureas and an increased risk of ventricular arrhythmias and sudden cardiac death [30].

Several observational studies, including the University Group Diabetes Programme and a sub-study of the UKPDS, have found evidence suggesting an increased cardiovascular risk associated with sulfonylureas. This elevated risk is particularly prominent in terms of cardiovascular mortality [29]. There were no signs of cardiovascular safety concerns in 15 carefully conducted long-term randomized controlled trials, spanning a duration of 72 weeks, that utilized sulfonylureas as part of the treatment approach for T2DM. These randomized control trials (RCTs) included notable trials such as ADVANCE and ADOPT that directly compared sulphonylureas with other active comparator medications [31,32]. However, it is worth noting that there remains limited data on cardiovascular incidents related to sulphonylureas, and the reporting of such incidents may be inconsistent and unreliable [29]. The meta-analysis conducted by Phung et al. [33] included 33 studies with a participant size of 1,325,446 individuals. The findings from these studies indicate that the use of sulphonylureas drugs as compared to other oral diabetes medications poses a significantly higher risk for cardiovascular death (RR 1.27; 95% CI 1.18-1.34) and composite cardiovascular events such as myocardial infarction, stroke, cardiovascular-related hospitalization or cardiovascular deaths (RR 1.10; 95% CI 1.04-1.16).

The CAROLINA trial demonstrated that the cardiovascular safety of glimepiride was comparable to that of linagliptin [29]. Therefore, the present black box warning for sulfonylureas on the risk of cardiovascular mortality needs to be reviewed by the US Food and Drug Administration (FDA). This would give all those at risk for cardiovascular disease, confidence to use modern sulphonylureas [34].

Adverse Effects

Regardless of blood glucose level, sulfonylureas stimulate insulin secretion. Consequently, hypoglycemia is the most common adverse effect associated with sulfonylureas and poses a significant safety concern [23,35]. Hypoglycemia is defined as a condition where blood glucose levels drop below 70 mg/dL, accompanied by symptoms such as sweating, shaking, restlessness, cognitive impairment, increased heart rate, and feelings of hunger [25,36,37]. For older individuals and those with renal or hepatic impairment, sulfonylureas should not be used [25].

Sulfonylureas often lead to weight gain; thus, they should not be prescribed to obese individuals [38,39]. Other typical adverse effects include dizziness, headaches, nausea, and diarrhea. Above all, sulfonylureas may be associated with the risk of ventricular arrhythmias and sudden cardiac death [30].

Thiazolidinediones

Thiazolidinediones (TZDs) are a group of medications that have been approved by the FDA for managing elevated blood glucose levels in individuals with T2DM, often used alongside other anti-diabetic drugs. The first TZD to obtain permission for use in the United States was troglitazone [40]. TZDs, including pioglitazone and rosiglitazone, exert their primary action through the activation of peroxisome proliferator-activated receptors [41]. This receptor plays a vital role in the regulation of carbohydrate, lipid, and protein metabolism. Over the years, these two widely prescribed TZDs have gained recognition as effective treatment options for glycemic control. They demonstrated significant improvements comparable to established agents, such as metformin and sulfonylureas [42]. By increasing adiponectin levels, TZDs enhance insulin sensitivity by encouraging the oxidation of fatty acids, lowering glucose generation, and lowering hepatic fatty acids and triglycerides [43]. TZDs commonly raise serum levels of low-density lipoprotein cholesterol; rosiglitazone has a more dramatic effect than pioglitazone [44]. 

*Cardiovascular Effects * 

A meta-analysis of RCTs revealed that rosiglitazone was found to be at a greater relative risk of myocardial infarction (1.58; 95% CI 1.14 to 2.20) and heart failure (1.48; 95% CI 1.01 to 2.18) than pioglitazone [45]. Troglitazone was removed from the US market in 2000 due to its hepatotoxicity. Furthermore, both rosiglitazone and pioglitazone have specific cautions regarding their association with congestive heart failure [46]. The frequency of cardiovascular comorbidities is a major factor in the rising burden of atrial fibrillation over time [47]. TZDs have been shown to have a positive impact on the atherosclerotic process; in particular, rosiglitazone and pioglitazone have been shown to lower inflammatory marker levels, slow the thickening of the intima of carotid vessels, and prevent restenosis after coronary stenting. Nevertheless, these advantages are accompanied by drawbacks such as fluid retention and exacerbation of heart failure [48]. The effects of pioglitazone on lipid metabolism may contribute to cardiovascular side effects. The PROactive and CHICAGO studies demonstrated that pioglitazone was associated with a statistically significant reduction in triglycerides (by 11% to 15%) and a significant increase in high-density lipoprotein (HDL) levels (by 9% to 14%) [49]. Pioglitazone enhances insulin resistance in T2DM in conjunction with the removal of harmful lipid metabolites and fat from muscle [50].

Adverse Effects

TZDs have few serious long-term adverse effects like weight gain and fluid retention causing exacerbation of heart failure and pulmonary edema [51].

DPP-4 inhibitors

DPP-4 inhibitors are a group of antidiabetic drugs that act by inhibiting the action of the enzyme dipeptidyl peptidase-4 (DPP-4), which is responsible for the breakdown of GLP-1, an incretin. By inhibition of this enzyme, the levels of incretins increase, leading to an increase in glucose-dependent insulin secretion, suppression of glucagon secretion, a delay in gastric emptying, and a decrease in caloric intake. This helps to control the hyperglycemia in diabetic patients [52].

Cardiovascular Effects

In a review by Zakaria et al., it was found that DPP-4 inhibitors had beneficial effects on the cardiovascular system by modulating the levels of vasoactive cardio-protectant peptides like B-type natriuretic peptide (BNP), stromal cell-derived factor 1α (SDF-1α) and substance P [53]. They also exert positive effects on the cardiovascular system through mechanisms like reducing hyperglycemia, reducing oxidative damage and inflammation, promoting tissue repair, and protecting the endothelial function, leading to better perfusion of the myocardium [53].

However, a meta-analysis showed that DPP-4 inhibitors resulted in a decrease in the risk for fatal and non-fatal stroke which was not significant (RR = 0.96, 95% CI: 0.85-1.08, I2 = 0%) while demonstrating a neutral effect on the risk for hospitalization due to unstable angina [54]. Three large, randomized-controlled trials showed that DPP-4 inhibitors did not have a significant effect on the risk of myocardial infarction or ischemic stroke while possibly increasing the risk of heart failure [55]. Another study linked the use of DPP-4 inhibitors to an increased rate of heart failure by showing that DPP-4 inhibitors activate the renin-angiotensin-aldosterone system (RAAS), which is involved in maintaining vascular tension and remodeling of the heart. Hence, long-term activation of RAAS can lead to sodium and water retention, and increase systemic vascular resistance and fibrosis. All these effects add to the workload of the heart in heart failure patients [56]. According to the results of a meta-analysis, DPP-4 inhibitors did not have any significant effect on the risk of atrial fibrillation (RR = 0.95, 95% CI: 0.78-1.17, I2 = 0%), while they were associated with a significant increase in the risk for atrial flutter (RR = 1.52, 95% CI: 1.03-2.24, I2 = 0%) [54].

Adverse Effects

The most common adverse effects of DPP-4 inhibitors include gastrointestinal effects like nausea, diarrhea, stomach ache, skin rash, and others like headache, sore throat, and upper respiratory tract infections [57].

GLP-1 agonists

Glucagon-like polypeptide-1 agonists are used for the treatment of T2D. They increase glycaemic control, thus preventing a sudden rise in blood glucose levels and maintaining healthy sugar homeostasis [58]. There are three main mechanisms that lower plasma glucose levels: (1) glucose-dependent insulinotropic actions, (2) decrease in glucagon hypersecretion, except during hypoglycemic episodes, and (3) decrease in gastric motility and delay in gastric emptying, which increases satiety. Glycolysis of blood glucose entering the beta cells of the pancreas causes the release of ATP, which inhibits the potassium ATP channels, causing depolarization, leading to an increase in intracellular Ca2+, which stimulates the secretion of insulin. The subsequent Ca2+ influx causes vesicular exocytosis, facilitating GLP-1 to be secreted into the bloodstream [59]. Thus, GLP-1 potently and quickly promotes insulin production [3]. GLP-1 enhances the sensation of fullness by activating specific receptors on the dendritic terminals of the vagus nerve, which innervate the digestive organs [60,61]. It also increases insulin sensitivity in peripheral cells.

Cardiovascular Effects

The cardiovascular effects of GLP-1 agonists have been studied in several RCTs. A meta-analysis of eight trials by Sattar et al. showed that the GLP-1 agonists were associated with reduced incidence of major adverse cardiovascular events (MACE) (HR 0.86; 95% CI 0.80-0.93) and hospital admission rates for heart failure (HR 0.89; 95% CI 0.82-0.98) [62]. According to another meta-analysis [63], GLP-1 agonists were associated with a reduction in the incidence of major adverse cardiac events (MACE), non-fatal myocardial infarction (MI), cardiovascular death, and hospitalizations for heart failure. The data suggests that GLP-1 agonists reduce systolic blood pressure by 2 to 6 mmHg. This could be the mechanism for the cardiovascular protection [64]. The effects on lipid profiles like reduction in low-density lipoprotein (LDL) levels, total cholesterol levels, and triglyceride levels can also explain the cardioprotective effect [65].

Adverse effects

According to various RCTs, it was found that exenatide and liraglutide produce more adverse drug reactions than albiglutide and lixisenatide [66]. Although the frequency of hypoglycemia was generally low in clinical trials testing GLP-1 receptor agonists in combination with sulphonylurea (with or without metformin) or insulin, it was higher compared to placebo [67,68]. In a meta-analysis [69], GI symptoms were found to be more frequent and severe at the beginning of the therapy, but with continuous administration, they decreased and disappeared. Nausea and diarrhea are common adverse events. Primitive GLP-1 agonist drugs like exenatide are known to cause chronic pancreatic injury leading to inflammatory changes in the cells but the newer GLP-1 drugs fail to show any similar effect [67,70]. GLP-1 agonists are known to cause hypersensitivity reactions like anaphylaxis, pruritus (the most common adverse injection site reaction), urticaria, and angioneurotic edema [67].

SGLT-2 inhibitors

SGLT-2 inhibitors work by inhibiting glucose reabsorption in the renal tubules through specifically targeting SGLT-2 receptors. This blocking action prevents glucose from being reabsorbed, resulting in an increased excretion of glucose in urine, which is referred to as glycosuria. As a result of this mechanism of action, SGLT-2 inhibitors not only reduce blood glucose levels in individuals with diabetes but also promote weight loss and offer cardiovascular benefits [3]. Numerous theories have been suggested to explain the positive impacts of SGLT-2 inhibitors on cardiovascular and renal well-being. These include enhancements in hemodynamics, decreased inflammation and fibrosis, antioxidative characteristics, as well as metabolic effects [71].

Cardiovascular Effects

According to a meta-analysis by Giugliano et al., SGLT-2 inhibitors decreased the incidence of MACE, non-fatal MI, cardiovascular death, and hospitalizations for heart failure [63]. These results were in agreement with those of a meta-analysis of six RCTs by McGuire et al. [72]. They reported that SGLT-2 inhibitors caused a reduction in major adverse cardiovascular events, hospitalizations for heart failure, and cardiovascular death. These meta-analyses included data from landmark trials like EMPA-REG OUTCOME, CANVAS program, and DECLARE-TIMI 58 trials [73-75].

Adverse Effects

The drawback of this particular drug is that it solely depends on renal health. Although it’s shown to be reno-protective, unhealthy kidneys would result in an improper filtration process and thus the drug would be ineffective [76]. SGLT-2 inhibitors increase the risk of genital mycotic infections due to increased urinary glucose excretion. Other notable risks of administering SGLT-2 inhibitors are urinary tract infection, ketoacidosis, and increased chance of amputation [77].

Insulin

Insulin is a hormone that plays an important role in metabolism, growth, and proliferation. Insulin acts through its tyrosine kinase receptor, consisting of the alpha and beta subunits. The signal transduction leads to the translocation of glucose transporters from the cytoplasm to the cell membrane, leading to increased cellular uptake of glucose. Through its effects on various tissues and organs, insulin promotes anabolic functions like glycogenesis, and lipogenesis, while inhibiting the breakdown of lipids and proteins [78].

Cardiovascular Effects

Insulin has been shown to have protective functions on the cardiovascular system through antioxidant and anti-inflammatory effects. Also, insulin is associated with coronary vasodilation action and improved myocardial perfusion [79]. A meta-analysis by Ruige showed a weak association between the levels of insulin and cardiovascular disease [80] while another meta-analysis showed no significant association of insulin on MACE, mortality, and heart failure [81].

Adverse Effects

Some of the most common adverse effects of insulin therapy include episodes of hypoglycemia, injection site reactions, lipodystrophy, weight gain, headache, and edema due to fluid retention [82].

Tirzepatide

Tirzepatide is a new medication that has the unique ability to activate both GLP-1 and gastric inhibitory polypeptide (GIP) receptors, leading to dual effects. The FDA has approved tirzepatide for the management of T2DM [83]. With a half-life of approximately 5 days, this drug is suitable for weekly subcutaneous administration [84].

Cardiovascular Effects

Many trials have been published on the efficacy of tirzepatide for the treatment of diabetes and obesity [85-87]. Some of them have also studied the cardiovascular safety of tirzepatide at different doses. According to a meta-analysis, tirzepatide was not associated with an increased risk of MACE, cardiovascular death, and MI [88].

Adverse Effects

Adverse events with tirzepatide are most commonly gastrointestinal-related such as nausea, vomiting, diarrhea, constipation, and dyspepsia. It can also cause acute pancreatitis, cholelithiasis, and cholecystitis [89].

## Conclusions

The findings of our review have significant implications for clinical practice, research, and the overall management of diabetes mellitus. By examining the cardiovascular effects associated with antidiabetic drugs, we gain valuable insights that can inform healthcare practitioners in their decision-making process when it comes to tailoring treatment regimens for individuals with diabetes. It is crucial for clinicians to understand the potential cardiovascular implications of various antidiabetic medications beyond just glycemic control, as addressing cardiovascular risk factors is equally important in managing diabetes effectively. By adopting a patient-centered approach that takes into account both glycemic control and cardiovascular well-being, healthcare providers can enhance patient outcomes. However, there are still gaps and areas of uncertainty pertaining to the effects of antidiabetic drugs on cardiovascular health that require further investigation.

In summary, the implications of the narrative review have far-reaching impacts on different aspects of healthcare, such as clinical practice, research priorities, regulations, public health policies, and patient involvement. By providing insights into the complex relationship between antidiabetic medications and cardiovascular well-being, this review aids in enhancing diabetes treatment strategies and enhancing overall patient results.
